# Global Challenges: Energy

**DOI:** 10.1002/gch2.1001

**Published:** 2015-09-21

**Authors:** Peter D. Lund

**Affiliations:** ^1^ Aalto University School of Science Espoo‐Otaniemi Finland

## Abstract

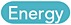

Energy is one of the most strategic resources and basic commodities necessary to support a modern industrialized society. Since the industrial revolution, access to commercial energy has enabled not only a better quality of life but also economic prosperity demonstrated by an increasing living standard. Abundant energy has spurred major technological and industrial development on our globe and has brought about great paradigm shifts, for example, in the way we travel. Societal development by and large has a close link to energy too. For example, the prosperous western way of life rests on almost unlimited access to energy supplies. Similarly, in the developing world, material scarcity is often connected to energy shortage.

Although energy may appear as a symbol for progress in a historical perspective, it is increasingly interlinked to the most serious global challenges of our time or even to the whole existence of the present societal structure. Energy is connected with key challenges such as climate change, environmental degradation, security, poverty, health, food production, agriculture, and water resources. Thus, when searching for better and more sustainable energy solutions, energy needs to be dealt with in a broader multidisciplinary framework considering these interconnections (Lund and Byrne, 2014; Sovacool, 2014).

The best publicized negative effect from energy is climate change associated with greenhouse gas emissions from the extensive use of fossil fuels, which still constitute the bulk of our energy production and on which many emerging growing economies in Asia and elsewhere rely. These emissions increase the global temperature, which in turn may damage our ecosystems and may lead to draughts, famine, extinction of species, human migration, and so on. Limiting these adverse effects to a tolerable level would require more than halving the global energy‐related emissions by the middle of this century.

The scientific community, in particular the Nobel Prize winning Intergovernmental Panel for Climate Change (IPCC) has brought unambiguous evidence between carbon emissions and climate change and has prepared options on how to mitigate the problem (Pachauri, 2012). Policymakers need to decide on the path forward and reach a global agreement on climate change mitigation. Finding solutions to complex issues such as energy‐induced climate change, or any global grand challenge, will require evidence‐based independent policy advice and foresight.

To understand the magnitude of the global challenge ahead, the term “energy revolution” is often used. Revolutions may, by definition, be associated with destruction and turmoil, but in the energy context, it could rather be about a Schumpeterian type of creative destruction leading to a better future. In the words of the UN World Commission on Environment and Development: “Sustainable development is development that meets the needs of the present without compromising the ability of future generations to meet their own needs”*.*

Different types of clean energy innovations and technology breakthroughs could have a positive impact on many of the global challenges and could even solve a number of crucial problems. For example, 1.3 billion people on our planet lack modern electricity services. The quest for development will lead to increasing demands for energy, which could also end up with competing for other critical resources or having conflicting impacts. Land re‐use through cultivation of biofuel crops may compete with food production for water and also lead to higher greenhouse gas emissions. Constructing more thermal power plants, which need much water for cooling, could compete for limited water resources.

Because energy is so crucial for development and prosperity, it is also subject to strong political influence and debate. Energy policies need increasingly to tackle with cross‐disciplinary issues linked to energy security, geopolitics, climate change, justice, poverty, energy markets, and public spending demonstrated by the European Union's recent Energy Union energy–climate policy package. Additional challenges will emerge from the growing urge to link energy policy to job creation and economic growth.

The challenge to build a sustainable path towards a low carbon society encompasses a major socio‐technical transition, which requires better understanding of the influence of social, technical, and economic factors, and their interactions (Foxon, 2011). The complexity in energy sustainability calls for strengthening of the science–policy dialogue to ensure that the decision‐makers possess the most up‐to‐date information on options and their far‐reaching consequences. Learned societies have an important function in pulling together objective information. In Europe, the European Academies' Science Advisory Council (EASAC) or the European Council of Academies of Applied Sciences, Technologies and Engineering (Euro‐CASE) serves as good example by providing independent science‐based and technology‐based policy advice to European institutions. Our intention is that *Global Challenges* will play a central role by providing a forum to disseminate advice and solutions to complex issues in energy.

*Global Challenges* offers a unique platform to pull intellectual capacity together to pursue a discussion on issues in energy. It will involve different stakeholders prone to multidisciplinary energy thinking. The scope of energy is by definition very broad. But rather than to limit the thematic range, we put emphasis on fresh and original contributions. These may include cutting‐edge research, high‐caliber analyses, unique experiences, or, new bold ideas to challenge conventional wisdom, among others. These could provide insight into new directions, practices, and solutions within the energy discipline itself or into energy relevant cross‐cutting themes across disciplines. We invite contributions which show strong relevance and broad applicability and which convey a policy message as well.

## References

[gch21001-bib-0001] Foxon, T.J. , 2011 A coevolutionary framework for analyzing a transition to a sustainable low carbon economy. Ecol. Econ. 70, 2258–2267.

[gch21001-bib-0002] Lund, P. , Byrne, J. , 2014 Energy and environment is defined by its cross‐disciplinary basis: an editorial essay. WIREs Energy Environ. 3, 1–2 doi: 10.1002/wene.104.

[gch21001-bib-0003] Pachauri, R.K. , 2012 The way forward in climate change mitigation. WIREs Energy Environ. 1, 3–8 doi: 10.1002/wene.36.

[gch21001-bib-0004] Sovacool, B.K. , 2014 Energy studies need social science. Nature 511, 529–530. 25079540 10.1038/511529a

